# Innate Immunity Effector Cells as Inflammatory Drivers of Cardiac Fibrosis

**DOI:** 10.3390/ijms21197165

**Published:** 2020-09-28

**Authors:** Denisa Baci, Annalisa Bosi, Luca Parisi, Giuseppe Buono, Lorenzo Mortara, Giuseppe Ambrosio, Antonino Bruno

**Affiliations:** 1 Immunology and General Pathology Laboratory, Department of Biotechnology and Life Sciences, University of Insubria, 21100 Varese, Italy; lorenzo.mortara@uninsubria.it; 2 Laboratory of Pharmacology, Department of Medicine and Surgery, University of Insubria, 21100 Varese, Italy; a.bosi@uninsubria.it; 3 Department of Biomedical, Surgical and Dental Sciences, School of Dentistry, University of Milan, 20122 Milan, Italy; luca.parisi@unimi.it; 4 Unit of Immunology, IRCCS MultiMedica, 20138 Milan, Italy; giuseppe.buono@multimedica.it; 5 Division of Cardiology, University of Perugia School of Medicine, 06123 Perugia, Italy; giuseppe.ambrosio@ospedale.perugia.it

**Keywords:** cardiac fibrosis, inflammation, neutrophils, macrophages, natural killer cells, eosinophils, mast cells

## Abstract

Despite relevant advances made in therapies for cardiovascular diseases (CVDs), they still represent the first cause of death worldwide. Cardiac fibrosis and excessive extracellular matrix (ECM) remodeling are common end-organ features in diseased hearts, leading to tissue stiffness, impaired myocardial functional, and progression to heart failure. Although fibrosis has been largely recognized to accompany and complicate various CVDs, events and mechanisms driving and governing fibrosis are still not entirely elucidated, and clinical interventions targeting cardiac fibrosis are not yet available. Immune cell types, both from innate and adaptive immunity, are involved not just in the classical response to pathogens, but they take an active part in “sterile” inflammation, in response to ischemia and other forms of injury. In this context, different cell types infiltrate the injured heart and release distinct pro-inflammatory cytokines that initiate the fibrotic response by triggering myofibroblast activation. The complex interplay between immune cells, fibroblasts, and other non-immune/host-derived cells is now considered as the major driving force of cardiac fibrosis. Here, we review and discuss the contribution of inflammatory cells of innate immunity, including neutrophils, macrophages, natural killer cells, eosinophils and mast cells, in modulating the myocardial microenvironment, by orchestrating the fibrogenic process in response to tissue injury. A better understanding of the time frame, sequences of events during immune cells infiltration, and their action in the injured inflammatory heart environment, may provide a rationale to design new and more efficacious therapeutic interventions to reduce cardiac fibrosis.

## 1. Introduction

Chronic pathological disorders, including cardiovascular diseases (CVDs) [1,2], neurodegenerative diseases [3,4], diabetes [5,6], metabolic syndrome [7,8], and cancer [9,10] share an inflammatory microenvironment as a hallmark [11]. A Common feature of these complex diseases is the activation of “sterile” inflammatory pathways, in which immune cells represent relevant effectors and drivers for the onset and progression of the disease [12], due to their extreme capability to reshape their phenotype [13], to functionally [14], metabolically [15,16] adapting to the surrounding environment. Among these inflammatory-mediated disorders, CVDs account as the leading cause of death worldwide. It is now clear that, besides cardiomyocytes and endothelial cells, altered immune response during CVDs impacts physiopathology, resolution, and/or progression of cardiac alterations. While some degree of immune cell activation is essential to promote response to injury and activation of tissue repair processes, unchecked activation may eventually lead to excess fibrosis, thus offsetting benefits.

A common final pathway of cardiac injury includes remodeling and fibrosis, contributing to heart failure (HF) development [17,18,19]. Indeed, in several distinct pathophysiological conditions, including cardiomyopathies, myocardial infarction (MI), pressure overload, and aging, the presence of cardiac fibrosis is a shared feature [20,21,22].

Fibrotic remodeling of the extracellular matrix (ECM) is a healing mechanism, absolutely necessary after myocardial injury. Yet, excess increase in myocardial fibrotic activity may result in stiffening of the myocardium, contributing to adverse outcome, as cardiac fibrosis is characterized by diastolic dysfunction [21], ventricular wall stiffening, reduced contractility, and impaired overall cardiac performance. Major features of cardiac fibrosis include altered and uncontrolled accumulation of ECM in the heart, due to its increased synthesis or decreased degradation [23,24]. Finally, myocardial interstitial fibrosis induces left ventricular dysfunction, leading to the development of heart failure.

Qualitative and quantitative modifications of the cardiac microenvironment following cardiac injury result from the crosstalk between a variety of cell types representing the “normal compartment” of the heart, such as fibroblasts, endothelial cells, inflammatory and immune cells, as well as soluble factors and the components of the ECM [25,26,27].

Here, we review and discuss the contribution of inflammatory cells from innate immunity to fibrosis onset and progression, focusing on inflammatory cells of innate immunity: neutrophils, macrophages, natural killer (NK) cells, eosinophils (EOs), and mast cells (MCs).

## 2. Host Non-Immune Cells and Fibrosis in the Heart

Cardiac cell populations and their mutual interactions sustain myocardial repair and regeneration following an insult. Cardiac fibroblasts account for 60–70% of the total number of heart cells and are key players in cardiac homeostasis maintenance [19]. Although cardiac fibroblasts are considered essential modulators of ECM remodeling, other cell types, including immune cells, vascular cells, and cardiomyocytes are implicated in cardiac fibrosis either directly (by secreting proteases or antiproteases), or indirectly (by modulating fibroblast phenotype). Long-term stress conditions or cardiomyocyte injury may trigger the release of fibrogenic cardiac mediators, leading to fibroblast activation and myofibroblast trans-differentiation. Phenotypically, myofibroblasts significantly express α-smooth muscle actin (α-SMA), ECM proteins including collagens, periostin, metalloproteinases (MMPs), and as compared to fibroblasts, are more contractile with active migratory, proliferative, and secretory properties [19,28,29]. While the pathophysiology of cardiac fibrosis is mostly attributed to excessive synthesis and accumulation of ECM proteins by activated myofibroblasts, their origin remains controversial [30,31].

Lineage tracing approaches suggest that resident fibroblasts are the primary source of activated myofibroblasts involved in cardiac fibrosis [32,33]. Depending on the type of the pathological stimuli, there are different transcriptional regulatory axes that control fibroblast plasticity: expression of surface membrane signals (integrins, syndecans, angiotensin II type 1 receptor), promotion of fibrogenic program through activation of myocardin-related transcription factor, and activation of intracellular signaling cascades (RhoA, Wnt/β-catenin, AKT signaling, FAK, TGF-β/Smad-4, MAPK) [25,34,35,36,37,38,39].

Other cell types, including bone marrow-derived cells, endothelial cells, and perivascular Gli1^+^ progenitors have been described as additional sources of myofibroblasts [40,41,42]. The release of inflammatory mediators drives homing and migration to the heart of bone marrow progenitor cells, which further transdifferentiate into myofibroblasts [43].

Vascular endothelial cells, the most abundant non-cardiomyocytes in heart, can also contribute to fibrosis by acting as a source of myofibroblasts, via endothelial–mesenchymal transition (EndoMT), acquiring a fibroblast-like phenotype [44,45]. In addition, endothelial cells activate fibroblasts by releasing pro-inflammatory cytokines/chemokines, pro-fibrotic mediators such as transforming growth factor-β1 (TGF-β1), fibroblast growth factors (FGFs), endothelin-1(ET-1) or by promoting the recruitment of immune cells through expression of adhesion molecules (ICAM-1) [46,47]. Similarly, pericytes may contribute to the fibrotic process through conversion to activated myofibroblasts, or through secretion of fibrogenic mediators such as platelet-derived growth factor-β (PDGF-β); however, these cells remain poorly characterized and their role remain less clear and documented [42].

Likewise, cardiomyocyte exhibit a fibrogenic profile and provide signals for macrophage recruitment, in response to stress. It has been reported that angiotensin II–induced stimulation of Ca^2+^/calmodulin-dependent protein kinase II (CaMKII ) contribute to priming and activation of the NOD-like pyrin domain-containing protein 3 (NLRP3) inflammasome in cardiomyocytes, which regulates the release of inflammatory cytokines IL-1β and IL-18 with fibrogenic activities [48]. Inflammasome activation in the heart can be triggered either by release of cell death-mediated danger-associated molecular patterns (DAMPs), or by stress signals (e.g., ATP, ROS, in response to non-ischemic conditions, such as pressure overload [49]. Targeting TGF-β receptor II signaling in cardiomyocytes has been associated with reduced maladaptive hypertrophy and development of cardiac fibrosis subjected to pressure overload [50].

As inflammation plays a crucial role in cardiac fibrosis, deciphering the role of immune cells on the cardiac microenvironment may provide novel targeted strategies against fibrotic remodeling.

## 3. Major Inflammatory Cytokines in Cardiac Fibrosis

A finely tuned balance of proinflammatory and profibrotic cytokines orchestrates the fate of bone marrow-derived heart-infiltrating cells and directly instructs the morpho-phenotype of the affected heart. While acute inflammation is characterized by fast resolution of the associated vascular changes, including edema and neutrophil infiltration, fibrosis results from persistent inflammatory state, where tissue remodeling and tissue-repair processes occur simultaneously [51,52,53,54]. Chronic fibrotic disorders share the steady and uncontrolled production of growth factors, proteolytic enzymes, angiogenic factors and fibrogenic cytokines, resulting in aberrant deposition of connective tissue components that progressively reshape and alter the normal tissue architecture [51,52,53,54].

TGF-β has been recognized as a major inducer and regulator of fibrosis in CVDs and cancers [55,56,57]. Following induction of inflammatory response, TGF-β acts by increasing the production of ECM components [58,59], together with enhanced mesenchymal cell proliferation, migration, and accumulation [60,61]. In the heart, TGF-β is largely produced by fibroblasts, macrophages, and T cells [62] although also cardiomyocytes are able to release TGF-β in response to angiotensin-II [63,64]. TGF-β induces the activation of fibroblasts and myofibroblasts producing inhibitor of metalloproteinases (TIMPs) [63,65,66] collagen I, collagen III, and fibronectin, thus supporting fibrosis, in a Smad3-dependent manner [67,68,69].

Inflammatory interleukins play a crucial role in the inflammatory-driven fibrosis in the heart. Interleukin-1 (IL-1) has been demonstrated to be largely increased in patients with chronic or decompensated HF [70], independent of the ischemic, hypertensive, idiopathic etiology [70]. Different mechanisms relating IL-1 to impaired systolic function have been proposed. For example, IL-1β has been shown to decrease the beta-adrenergic responsiveness of L-type calcium channels in a cAMP-independent manner [71]. Also, IL-1β has been associated with a reduced expression of genes involved in the regulation of calcium homeostasis, including phospholamban, sarcoplasmic reticulum calcium ATPase [72,73]. IL-1 is largely produced during the inflammatory phase of cardiac repair [74,75]. Beside its pro-inflammatory functions, IL-1 induces increased synthesis and production of MMPs by cardiac fibroblasts [76,77]. Interestingly, some members belonging to the IL-1 family, such as IL-33 and ST2, are endowed with favorable cardiac effects [78]. IL-33 and ST2 regulate monocyte infiltration in the heart and protect from hypertrophy and fibrosis [78].

Another crucial cytokine, also found increased during fibrosis is IL- 4, playing an active role in the fibrogenic process by activating fibroblasts and collagen synthesis [79]. Similarly, IL-6 promotes interstitial cardiac fibrosis via TGF-β, enhancing cardiac fibroblast proliferation, collagen production and deposition [80,81].

## 4. Innate Immunity Cells Contribution to Cardiac Fibrosis

The inflammatory process, as a result of aberrant phenotype and functions of the innate and adaptive immune system, is now recognized as a relevant hallmark in CVDs. Clinical and preclinical studies have established that cardiac fibrosis is associated with inflammation, characterized by a rapid, dynamic, and continuous innate immune response. The presence of innate immunity effector cells, most of them being macrophages, neutrophils, NK cells, eosinophils, and MCs, has prompted research in investigating their role and function in cardiac fibrosis.

### 4.1. Neutrophils

Neutrophils are the most abundant white cell type in human blood, representing 60–70% of circulating leukocytes [82]. Neutrophils are short-lived cells, with a median lifespan of 4.3 h–5.4 days in humans [83,84,85]. Although traditionally considered as a homogeneous population, accumulating evidence has demonstrated their heterogeneity showing the existence of peculiar subsets with phenotypic and functional differences able to display different roles both in homeostatic and pathological conditions [86,87]. Several studies pointed out the central role of neutrophils and their mediators in CVDs, including atherosclerosis, cardiac hypertrophy and fibrosis [88].

**Figure 1:**
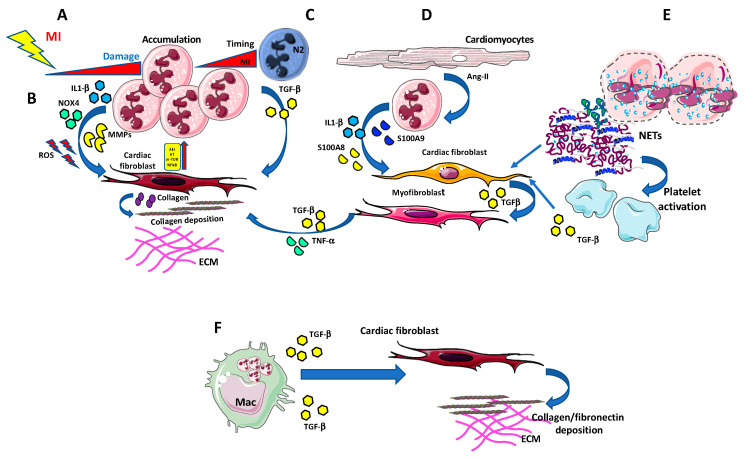
Neutrophils in cardiac fibrosis. (**A**) Neutrophils massively infiltrate the infarcted area in the first few hours being the earliest detectable immune population following ischemia onset. (**B**) Neutrophils can release large amount of pro-fibrotic agents, such as Interleukin-1 (IL-1)β, NOX4, and metalloproteinases (MMPs), which, along with the generation of ROS, instruct cardiac fibroblasts to produce collagen and support collagen deposition, in an Akt/mTOR, NFκB-dependent manner. (**C**) N2-like neutrophils accumulate in the infarcted area and release TGF-β, thus favouring collagen production and deposition by cardiac fibroblasts. (**D**) Injured cardiomyocytes are able to activate pro-fibrotic/pro inflammatory/TGF-β producing myofibroblasts, using IL-1β, S100A8, and S100a9 producing neutrophils, as bystander cells. (**E**) Neutrophils can also impact MI and fibrosis by releasing extracellular traps (NETs). NETs promote the recruitment and activation of platelets that are a relevant source of TGF-β, thus indirectly supporting fibrosis. (**F**) Apoptotic neutrophil elimination by macrophages represent a crucial anti-inflammatory and pro-resolving signal itself. Apoptotic neutrophils induce anti-inflammatory mediators, including TGF-β, IL-10, and resolvins, which are pivotal in driving pro-resolving microenvironment.

Neutrophils play a major role in injury resolution, as they activate and accumulate within minutes following acute myocardial injury; they are responsible for debris removal as well as reparative response orchestration. As the predominant phagocyte cells in blood, neutrophils are the first immune cell type infiltrating the inflammatory site in response to release of alarmins, following cardiac insult, thus being the earliest detectable immune population following ischemia onset [89] (Figure 1A).

Neutrophils appear to play a double-edged role in cardiac fibrosis related to pathological conditions such as myocardial infarction, or myocarditis [90,91,92]. Neutrophil counts and hyperactivation have been considered as predictor of cardiac tissues remodeling and adverse clinical outcomes in the acute inflammatory phase after MI [93,94,95].

Once migrated into the heart tissues, neutrophils become activated and accumulate in the infarcted border zone, releasing high levels of reactive oxygen species (ROS) and soluble mediators, including inflammatory cytokines and proteolytic enzymes (such metalloproteinases) which exacerbate tissue injury [94,96] (Figure 1B). ROS promote myocardial fibrosis, thus providing a link between cardiac fibrosis and neutrophils. NADPH oxidase 4 (Nox4)-derived ROS regulate collagen synthesis in cardiac fibroblast through AT-1 pathway [97] and by activating Akt/mTOR and NFκB signaling. Nox4 inhibition attenuated cardiac remodeling [98] (Figure 1B).

Following cardiac injury, neutrophils release IL-1β through NLRP3 activation [99], and upregulate IL-1R in cardiomyocytes (Figure 1B) and cardiac fibroblasts, promoting myocardial apoptosis, fibrosis and inflammation. Recently it was suggested that calcium-sensing receptor on neutrophil orchestrate IL-1β release via NLRP3 inflammasome activation [100].

The contribution of neutrophils to cardiac fibrosis, however, remains to be fully elucidated. In this respect, Horckmans et al., have demonstrated that neutrophil-depleted mice subjected to acute myocardial infarction (aMI) actually showed worsening cardiac function and increased fibrosis compared to the wild-type counterpart [89]. Indeed, neutrophil depletion in mice induced the reduction of splenic Ly6C^high^ monocytes and the parallel increase of macrophage proliferation within the infarct area [89]. Moreover, 7 days post aMI, a significant down-regulation of IL-12, TNF-α, IFN-γ, IL-10, IL-1β (M1 markers), and up-regulation of CX3CR1, Arginase, Ym1, and IL-4 (M2 markers) was observed in Ly6G-depleted versus control mice, suggesting that neutrophils are crucial to inducing macrophage polarization towards the M1 phenotype [89]. Moreover, they found that macrophages in neutrophil-depleted mice showed impaired scavenging ability to clear apoptotic cardiomyocytes due to the reduction of the phagocytosis receptor MertK, resulting in increase of inflammation [89].

The peculiar role of neutrophils following MI was also highlighted by Daseke et al., that have recently explored neutrophil contribution to myocardial infarction [92]. Indeed, by performing an aptamer proteomics of cardiac neutrophils isolated from the infarct region, they demonstrated that neutrophils undergo a polarization process from N1 to N2 phenotype over the first week of MI [92] (Figure 1C). In particular, Daseke et al., showed that immediately after MI, neutrophils express pro-inflammatory genes, displaying a high degranulation profile characterized by a high MMP activity, yet after three days they exhibited a reparative signature, characterized by up-regulation of fibronectin, galectin-3, and fibrinogen expression, all molecules capable to contribute to ECM reorganization [92] (Figure 1C).

At the molecular level, it has been shown that neutrophils, stimulated by angiotensin II, release S100a8/S100a9 that can bind several receptors, including the toll-like receptor 4 and more importantly, RAGE which is expressed by fibroblasts [101]. S100a8/S100a9-RAGE binding induced phosphorylation of NFκB, activating inflammatory responses in cardiac fibroblasts and promoting cytokines secretion and cell migration, without affecting the proliferation and differentiation into myofibroblast, resulting in increased cardiac fibrosis [101].

Neutrophil-dependent upregulation of IL-1β enhances MMP9 activity, and its binding to IL-1R stimulate a matrix-degrading program in fibroblasts, while delaying myofibroblast conversion [102,103] (Figure 1D). This was correlated with excessive fibrosis and increased collagen content induced by the neutrophil’s depletion [104]. Thus, strategies to block acute neutrophil-driven inflammation should be carefully evaluated since they could enhance cardiac fibrosis and remodeling [104].

Apoptotic neutrophil elimination by macrophages represent a crucial anti-inflammatory and pro-resolving signal itself [105,106]. Apoptotic neutrophils induce anti-inflammatory mediators, including TGF-β, IL-10 and resolvins [107,108] that are pivotal in driving pro-resolving microenvironment (Figure 1F). However, the tissue repair response may promote a fibrogenic macrophage phenotype [109], since TGF-β is the master regulator of collagen deposition and fibrosis [110,111].

Neutrophils can also impact MI and fibrosis by releasing extracellular traps (NETs) [112,113]. NETs are constituted by DNA and antimicrobial proteins that generate a complex network that entrap extracellular pathogens, favoring their elimination [112,113]. Apart from their antimicrobial host defense activities, NETosis occurs also under non-infectious conditions, such as hypoxia, myocardial ischemia/reperfusion, and myocarditis. Fibroblasts exposed to NETs in vitro have been reported to support the ability of fibroblasts to transdifferentiate, enhancing their ability to proliferate, migrate and producing collagen.

Accordingly, NETs were found in proximity to α-SMA-positive fibroblasts and expressed IL-17 in tissue sections from patients with fibrotic intestinal lung disease [114]. NETs promote recruitment and activation of platelets [115] that are a significant source of TGF-β, so indirectly promoting fibrosis. NETs were found to stimulate in vitro macrophage polarization toward a reparative phenotype. Recently, it has been reported that NETs drive in vitro macrophage polarization toward an anti-inflammatory, pro-fibrotic M2 phenotype.

Finally, NETs seem to have a crucial role in initiating excessive deposition of collagen and fibrosis, thus likely contributing to heart failure [89,112,114,116,117]. Future studies will be required to better understand the mechanisms linking neutrophil-initiated inflammation to cardiac fibrosis.

### 4.2. Macrophages

Macrophages are heterogeneous, highly plastic cells of the innate immune system, involved in the primary response against microorganisms, inflammation, homeostasis, and tissue regeneration/repair [118]. Tissue regeneration is necessary for the development of an efficient healing process, and uncontrolled regulation of these mechanisms leads to fibrotic and scarring responses. Macrophages have been recently identified as critical regulators of fibrosis in several organs, including lung, liver, and heart [119,120,121,122]. In the heart, diverse macrophage populations, derived both from resident tissue macrophages and bone marrow progenitors, cooperate in the initiation, maintenance and resolution of the fibrogenic response [123,124,125,126].

**Figure 2:**
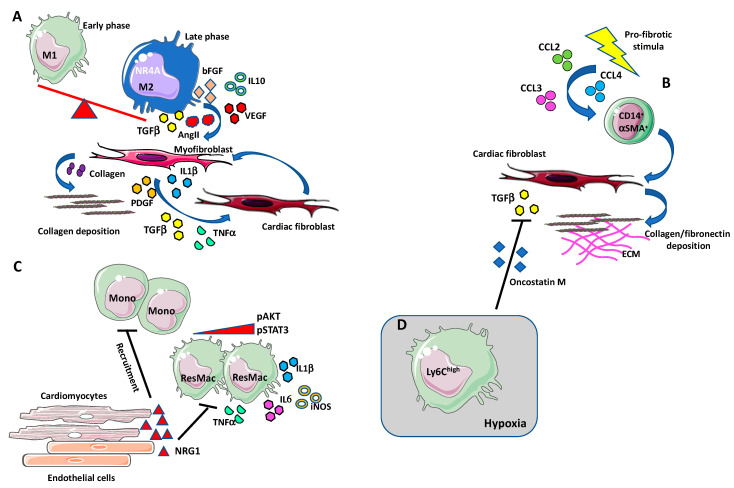
Macrophages in cardiac fibrosis. (**A**) During the first phase of tissue inflammation, macrophages acquire a “classically activated” (M1-like) state. In the later phase, macrophages switch into a reparative phenotype, producing anti-inflammatory cytokines, chemokines, and growth factors such as IL-10, TGF-β, VEGF, angiotensin II, bFGF, and PDGF [114,115]. The transition to this reparative state seems to be induced via nuclear receptor subfamily 4 group A member 1 (NR4A1). M2-like macrophages also produce the pro-fibrotic agent TGF-β that induce collagen secreting and collagen stabilizing myofibroblasts. High numbers of macrophages accumulate in the damaged heart, localizing in proximity to myofibroblasts, that, by producing TGF-β, angiotensin II, PDGF, TNFα, and IL-1β, stimulate induce the differentiation of cardiac fibroblasts into myofibroblasts in an autocrine manner. (**B**) Some of the cardiac infiltrating fibroblast can originate from a circulating monocytic CD14^+^ cell subset, termed fibrocytes. Under profibrotic stimulation, these cells have been shown to increase the expression of ECM components, such as collagen and fibronectin, and of the mature myofibroblast marker α-SMA. Increased number of circulating fibrocyte has been observed during cardiac fibrosis, in response to the augmented circulating levels of MCP-1/CCL2, CCL4, and CCL3. (**C**) Neoregulin-1 (NRG-1) can exert antifibrotic and anti-inflammatory effects acting on macrophages in an ErbB4-mediated manner. After fibrotic stimuli, NRG-1, released from damaged endothelial cells in the endocardium, activates ErB4 and downregulates the PI3K/Akt pathway and the phosphorylation of STAT3 thus reducing the release of proinflammatory mediators such as IL-1β, iNOS, IL-6, and TNF-α. The activation of ErbB4 results in the reduction of new monocytes recruitment and suppression of the inflammatory state. (**D**) Ly6C^high^ macrophages infiltrate in hypoxic areas in a hypoxia-inducible factor (HIF-1α)-dependent manner and inhibits TGF-β cardiac fibroblast activation by the release of oncostatin M (OSM).

Depletion or alteration of macrophages, either in the initial proinflammatory or during the final regenerative phases, have been associated with important consequences for cardiac functional recovery [127,128]. Given macrophages plasticity, they can exert both profibrotic and anti-fibrotic activities [128]. During the first phase of tissue inflammation, macrophages acquire a “classically activated” (M1-like) state; they express the chemokine receptor CCR2 and are associated with the release of proinflammatory cytokines (e.g., IL-1β, IL-6, TNF-α) and with phagocytic and proteolytic activity, that exacerbates the inflammatory process [129]. In the later phase, macrophages switch into a reparative phenotype, producing anti-inflammatory cytokines, chemokines, and growth factors such as IL-10, TGF-β, VEGF, angiotensin II, FGF, and PDGF [130,131]. The transition to this reparative state seems to be induced via nuclear receptor subfamily 4 group A member 1 (NR4A1) [127] (Figure 2A). On the other hand, “alternatively activated” (M2-like) macrophages regulate the degradation of extracellular matrix components through the release of MMPs, and secrete TGF-β to stimulate the activation of cardiac fibroblasts to collagen-secreting myofibroblasts, which are primarily involved in scar formation and cardiac fibrosis [132] (Figure 2A).

High number of macrophages accumulates at the site where cardiac injury occurs, in close proximity to myofibroblasts [133]. In turn, myofibroblasts are able to release cytokines such as TGF-β, angiotensin II, PDGF, TNFα, and IL-1β, to stimulate differentiation of cardiac fibroblasts into myofibroblasts in an autocrine manner (Figure 2A). Simões and colleagues showed that macrophages may directly contribute to scar collagen production in zebrafish and mouse models of heart injury [134]. These data are in line with the hypothesis on the hematopoietic origin of myofibroblast [135,136]. Some of the cardiac infiltrating fibroblasts appear to originate from a circulating monocytic subset of CD14^+^ cells, also called “fibrocytes”, that have been identified in human subjects and endowed with stemness features [137,138] (Figure 2B). Under profibrotic stimulation, these cells have been shown to increase the expression of ECM components, such as collagen and fibronectin, and of the mature myofibroblast marker α-SMA in in vivo and in vitro experimental models [139,140,141,142]. Increased number of circulating fibrocytes have been observed during cardiac fibrosis, in response to the augmented circulating levels of MCP-1/CCL2, CCL4, and CCL3 [136,138,143,144,145] (Figure 2B). This suggests that the stimulation of chemokine receptors contribute to the pro-fibrotic pathway, causing a recruitment of myofibroblast progenitors to the injured site. Interestingly, CCL2 overexpression has been demonstrated to correlate with increased macrophage infiltration, dilatative remodeling, and fibrosis, in murine cardiac muscle [146], and depletion of CCR2^+^ macrophages resulted in smaller infarct size [147]. Likewise, CCL2-null mice appeared to be protected against mineralocorticoid-induced cardiac fibrosis [148]. In this scenario, CCL2 appear as a master regulator of fibrosis via recruitment of monocytic-derived fibrocytes [148].

In contrast to the peripheral blood monocytic populations recruited, macrophages residing in the cavity of the pericardial space and expressing the transcription factor Gata6, have been observed to directly migrate into the injured site and prevent fibrosis, improving functional cardiac recovery after ischemic injury [149].

Mechanisms that influence macrophage response are represented by efferocytosis, DAMPs production, hypoxia, and ECM remodeling [150]. Deficiency of the phagocytic receptor MertK was shown to reduce the clearance of apoptotic cells by macrophages leading to delayed inflammation resolution after MI, adverse remodeling, and impaired cardiac function [151]. MertK receptor along with the neutrophil gelatinase-associated lipocalin (NGAL) was identified as key inducer of macrophages with high efferocytosis capacity [89,151]. However, ongoing cardiomyocyte death—a feature of severe infarction or prolonged ischemic injury—promotes a pro-inflammatory macrophage phenotype with less efferocytotic activity [152], namely a macrophage committed to eliminate apoptotic neutrophil and dead cell debris in response to heart injury [153,154]. Interestingly, following the engulfment with dead cells, macrophages acquire pro-regenerative/fibrotic feature by secreting TGF-β and IL-10, with reduced IL-12 release [153,154]. Therefore, persistence of dead cells debris leads to an increased secretion of DAMPs, which contribute to maintain continuous inflammatory signals acting on TLR4, resulting in reduction of infarct size, decreased activation of NF-κB and downregulation of IL-1β, CCL2/MCP-1, and IL-6 expression [155]. TLR4/TLR6-IRAK4/1 signaling was reported to enhance cardiac oxidative stress and subsequently activate NLRP3 inflammasome, thus inducing the cleavage and release of IL-1β and promoting cardiac fibrosis in rat [156].

Proteolytic release of the endogenous activator of epidermal growth factor receptors (ErbBs), neuregulin-1 (NRG-1), is involved in the adaptation of cardiovascular system to stress [157,158]. In diabetic rats with chronic heart failure, NRG-1 was observed to reverse myocardial interstitial fibrosis [159]. NRG-1 seems to exert antifibrotic and anti-inflammatory effects acting on macrophages in an ErbB4-mediated manner [160,161,162]. After fibrotic stimuli, NRG-1, released from damaged endothelial cells in the endocardium, activates ErB4 and downregulates the PI3K/Akt pathway and the phosphorylation of STAT3 thus reducing the release of proinflammatory mediators such as IL-1β, iNOS, IL-6 and TNF-α (Figure 2C). The activation of ErbB4 results in the reduction of new monocytes recruitment and suppression of the inflammatory state [162] (Figure 2C).

Another critical regulator of cardiac fibrosis is represented by hypoxia [163] (Figure 2D). In a murine model of cardiac remodeling, Ly6C^high^ macrophages infiltrate in hypoxic areas in a hypoxia-inducible factor (HIF-1α)-dependent manner, and inhibit TGF-β cardiac fibroblast activation via release of oncostatin M (OSM) [163]. OSM appear to have cardio protective effects on cardiomyocytes and fibroblast activating ERK signaling [164,165] (Figure 2D). In addition, the secretion of OSM induces in cardiomyocytes the production of the essential trafficking regulator of macrophages, the regenerating islet-derived 3β (Reg3β), causing a positive feedback loop which controls accumulation of macrophages in the heart [164].

Macrophages contribute to ECM remodeling process by secreting MMPs, the enzymes responsible for the degradation of matrix architecture. Several studies reported MMPs as regulators of macrophages phenotype and functions [103]. Surprisingly, high levels of MMP9 were described to associate to the surface of activated macrophages stimulating the cleavage of the α_2_-integrin protein (CD18) in lung, suggesting a mechanism of macrophage interaction with ECM components [166]. Since high level of MMP9 has been reported in patients, associated with adverse ventricular remodeling [167], and upregulation of MMP14 was correlated with reduced post MI survival and cardiac function in mice [168], it might be hypothesized the involvement of a similar mechanism. Moreover, the provisional fibrin structure exhibits growth factor-binding capacity [169]. Thus, the interaction with released PDGF, VEGF, and TGF-β may modulate macrophages functions and regulate the activation of myofibroblast (Figure 2A).

### 4.3. NK Cells

NK cells have been classified as type-I innate lymphoid cells [170]. They are large granular lymphocytes of innate immunity, involved in the recognition and elimination of virus-infected and malignant-transformed cells [171]. Based on surface antigen expression of CD56 (Neural cell adhesion molecule-NCAM) and CD16 (FcγRIIIa), two main NK cell subsets have been characterized. CD56^dim^CD16^+^ NK cells (90–95% of peripheral blood NKs) have cytolytic activities, by producing perforin, granzyme, and exerting antibody-dependent cellular cytotoxicity (ADCC) [171]. CD56^bright^CD16^-^ NKs (5–10% of peripheral blood NKs) acts via IFN-γ and TNF-α secretion [171]. Beside their role in tumor immunosurveillance, NK cells acquire “builder” rather than “killer” activities in specific pathophysiological contexts. Within the developing decidua, NK cells account for 50% of lymphocytes, acquire the CD56^superbright^CD16^-^ subsets and produce VEGF, PlGF, and CXCL8/IL-8 [172,173]. These decidual-NK cells (dNKs) are necessary for the formation of spiral artery that provide oxygen and nutrients to the developing fetus [172,173]. In different solid cancers, including non-small cell lung cancer (NSCLC) [174] and colorectal cancer (CRC) [175], NK cell have been demonstrated to acquire pro-angiogenic phenotype and function, described as CD56^bright^CD16^-^CD9^+^CD49a^+^VEGF^+^CXCL-8^+^IFN-γ ^low^, similar to dNKs [176,177,178,179,180].

NK cells account for over 1% of cardiac lymphocytes and participate to the regulation of process involved in cardiac diseases [181]. In patients with coronary artery diseases (CAD), NK cells have been found to be decreased in number and function, but not altered in their phenotype [182]. Also, NK cell deficit was found more frequently in patients with acute coronary syndrome [183].

NK cells have been reported to play a crucial role in repairing damaged tissues and maintaining tissue homeostasis [184]. NK cells have been reported to exhibit protective activities against acute viral pathogens such as CVB and murine cytomegalovirus induced myocarditis in vivo [185].

Studies in murine models and humans demonstrated NK cell capability to resolve and prevent fibrosis in the liver. Mice in which fibrosis has been experimentally induced by carbon tetrachloride experienced reduced severity of diseases following NK cell transfer. NK cells have been extensively demonstrated to be able to target activated fibroblasts, the major orchestrators of fibrosis.

**Figure 3:**
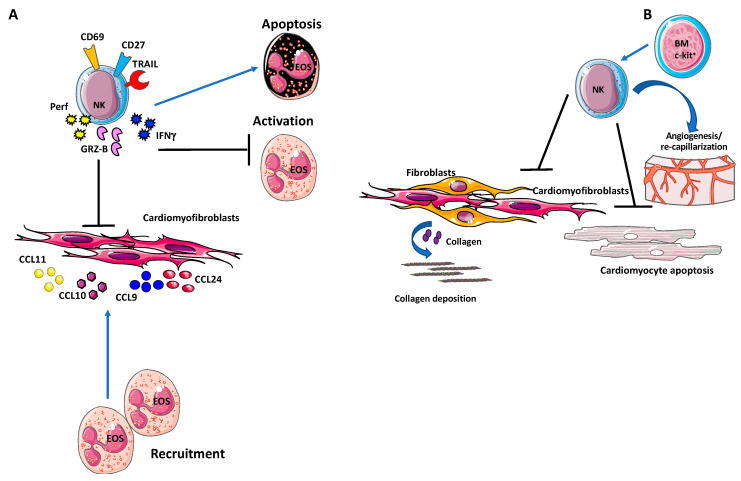
Natural killer (NK) cells in cardiac fibrosis. (**A**) Activated NK cells have been found to accumulate in the heart and release granzyme-B and IFN-γ, along with enhanced expression of CD69, TRAIL, and CD27 activation markers. This NK cell hyperactivation result in decreased cardiac fibrosis by inhibiting eosinophil activation and inducing eosinophil apoptosis, within an anti-inflammatory microenvironment. (**B**) Following myocardial infarction, expansion of NK cells from c-Kit^+^ bone marrow cells have been reported to protect the heart by reducing cardiomyocyte apoptosis [186], deposition of collagen and subsequent fibrosis, and by promoting neovascularization.

In an experimental model of acute myocarditis (EAM), it has been found that activated NK cells accumulate in the heart and release perforin, granzyme-B and IFNγ, along with enhanced expression of CD69, TRAIL, and CD27 activation markers (Figure 3A). This NK cell hyperactivation results in decreased cardiac fibrosis by inhibiting eosinophil activation and inducing eosinophil apoptosis, within an anti-inflammatory microenvironment (Figure 3A).

Depletion of NK cells, by anti-asialo GM1 antibody resulted in increased severity of myocarditis, augmented collagen deposition and elevated fibrosis, with a 10-fold increase of SSC^high^Ly6G^low^SiglecF^+^ eosinophil infiltration.

NK cells also limit eosinophil infiltration in the heart, by altering eosinophil-related chemokine production, eotaxin 1 (CCL11), eotaxin 2 (CCL24), CXCL9, and CXCL10, by resident cardiac fibroblasts (Figure 3A).

Following myocardial infarction, expansion of NK cells from c-Kit^+^ bone marrow cells has been reported to protect the heart by reducing cardiomyocyte apoptosis [186], deposition of collagen and subsequent fibrosis [186], and by promoting neovascularization [187] (Figure 3B).

This clearly place NK cells as possible cellular effectors to develop cell therapy strategy to prevent cardiac fibrosis.

### 4.4. Eosinophils

Eosinophils (EOs) can cause extensive damage to cells and tissues, but are also central in wound healing, tissue repair, and fibrosis [188]. Different pathological conditions associated eosinophilia, such as asthma [189], eosinophil myalgia syndrome, eosinophilic endomyocardial fibrosis [190], idiopathic pulmonary fibrosis [190], scleroderma [190], and eosinophilic esophagitis, share aberrant fibrosis as hallmark. Activated EOs infiltrate the site of inflammation and release degranulation proteins, cytokines, and growth factors, thereby promoting tissue injury and remodeling.

Several studies suggest significant roles for EOs in cardiac inflammation, fibrosis, and heart failure [191,192,193].

**Figure 4:**
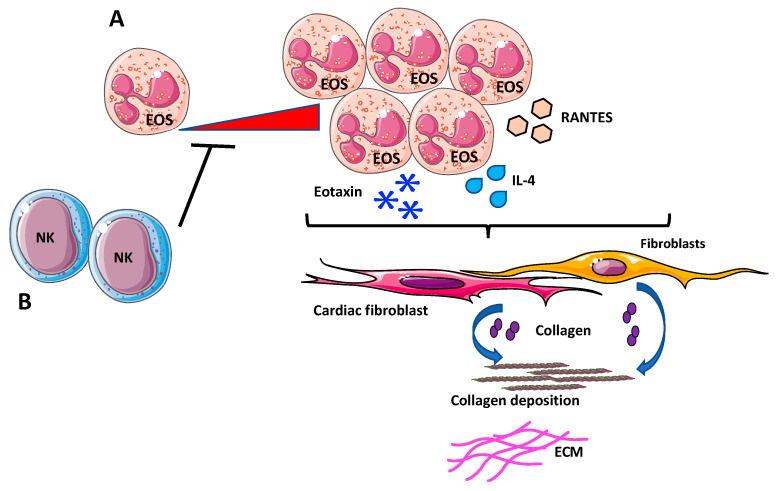
Eosinophils (EOs) in cardiac fibrosis. (**A**) Increased infiltrations of EOs, overexpressing chemokines and cytokines involved in innate and adaptive immunity, such as IL-4, eotaxin, and RANTES has been reported to support fibrosis, by activating cardiac fibroblasts to release and deposit collagen. (**B**) Depletion of NK cells generated a pro-eosinophilic environment, as showed by the high increase of cardiac-infiltrating EOs in vivo that was correlated with increased fibrosis.

Patients with elevated eosinophilia have been reported to be at higher risk to develop cardiac complications [192,194], via IL-4 secretions, driving the progression of myocarditis to inflammatory dilated cardiomyopathy [192]. An animal study employing a spontaneous mouse mutant with a hyper-eosinophilic phenotype, showed that the development of heart disease associates with increased infiltrations of EOs, overexpression of chemokines and cytokines involved in innate and adaptive immunity including IL-4, eotaxin, and RANTES [194], which in turn activate cardiac fibroblasts to release and deposit collagen [192] (Figure 4A).

Additionally, evidence suggests that EOs act like fibrotic mediators directly or through interactions with NK cells. In a mouse model of experimental autoimmune myocarditis, depletion of NK cells generates a pro-eosinophilic environment, as showed by the high increase of cardiac-infiltrating eosinophils in vivo that was correlated with enhanced fibrosis [191] (Figure 4B).

Although evidence supports the hypothesis that EOs are important players in cardiac fibrosis, further studies in suitable in vivo and ex vivo experimental models are needed. In this regard, employing eosinophil-deficient and hyper eosinophilic mouse models, Diny et al., showed that EOs drive progression of myocarditis to inflammatory dilated cardiomyopathy through IL-4 [194].

Further experimental studies employing EOs depleted animal models might address molecular mechanisms involved in fibrogenic responses.

### 4.5. Mast Cells

Mast cells (MCs) are best known for their pleiotropic phenotypes and functions in inflammatory processes such as allergy, infection, and tissue injury. In addition, other roles for MCs in several disorders, including cardiac pathologies have been widely documented [195,196]. Being highly dependent on the microenvironment in which they reside, MCs respond to inflammatory cytokines/chemokines, bacterial/viral products and DAMPs, through multiple receptors, including TLRs and the IL-33 receptor ST2 [197]. Primed MCs degranulate and secrete a plethora of mediators, including proteases, lysosomal enzymes, pro/anti-inflammatory cytokines, as well as factors with pro- and anti-fibrotic activities [196,198,199]. Given the MCs capability to produce both pro- and anti-fibrotic mediators, several studies have reported controversial results and described MCs divergent functions, including detrimental, neutral, or protective activities of MCs in cardiac remodeling and fibrosis. Discrepancies in the literature could be related to limited human in vitro data and existing differences of MCs content between animal models [198,199].

**Figure 5:**
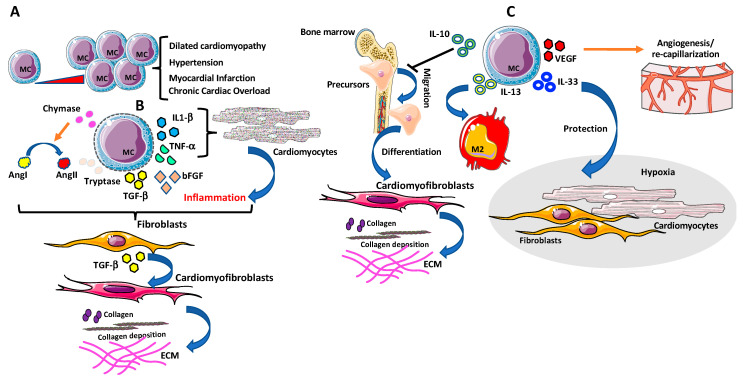
Mast cells (MCs) in cardiac fibrosis. (**A**) Accumulation of MCs in cardiac tissues has been observed in several cardiovascular diseases (CVDs). (**B**) MCs secrete several pro-fibrogenic factors such as bFGF, chymase and tryptase that have been long linked with cardiac fibrosis since can trigger fibroblasts activation directly or by promoting AngII, and TGF-β1. MCs degranulation- derived inflammatory cytokines, including TNF-α, IL-1β, can also drive fibrotic remodeling of the heart, via TGF-β-producing fibroblasts and by enhancing collagen production and deposition in cardiac fibroblasts. (**C**) MCs also act as anti-fibrotic mediators and anti-inflammatory cytokines/chemokines. MCs can produce IL-10, IL-13, and IL-33, known as potent inhibitors of the fibrotic signaling by blocking bone marrow fibroblast precursor cell migration in the heart and their differentiation towards myofibroblasts, triggering cardiac tissue resident macrophage to display M2 anti-fibrotic phenotype and attenuating tissue-remodeling and reduces fibrosis after cardiac injury, respectively. Finally, vascular endothelial growth factor (VEGF)-producing MCs promote re-capillarization of the cardiac tissue and reduce fibrosis.

Under physiological conditions, low number of MCs reside within the myocardium. Notably, increased number of MCs have been first associated with cardiac fibrosis more than 50 years ago in human hearts with endocardial fibrosis [200] (Figure 5A).

Strengthening this relationship were other studies reporting high numbers of MCs in areas with collagen deposition, in close proximity to the remodeling myocardium as described in explanted human hearts with dilated cardiomyopathy and in animal models of experimentally induced hypertension, myocardial infarction, and chronic cardiac volume overload [201,202,203,204,205,206,207] (Figure 5A). Accordingly, MCs deficient mice models showed reduced inflammatory responses and myocardial damage following a local insult [208]. MCs secrete fibrogenic products such as bFGF, chymase, and tryptase that have been long linked with cardiac fibrosis as they can trigger fibroblast activation directly or by promoting AngII, and TGFβ-1 [209]. MC degranulation-derived inflammatory cytokines including TNF-α, IL-1β [198] can also drive fibrotic remodeling of the heart [198] (Figure 5B). Several in vivo studies in different animal models reported reduced collagen deposition, after inhibition of MCs degranulation [210].

In a rat model of spontaneous hypertension, inhibition of MCs degranulation prevented collagen synthesis and fibrosis by normalizing IL-6 and increasing IL-10 levels, thus identifying new MCs mechanisms independent of degranulation [211].

MCs also serve as sources of anti-fibrotic mediators and anti-inflammatory cytokines/chemokines. Depending on the type of stimulus, MCs produce IL-10, IL-13, and IL-33, known as potent inhibitors of the fibrotic signaling [43] (Figure 5C).

IL-10 has been reported to limit fibrosis in a murine model of pressure overload (PO)-induced cardiac fibrosis, by blocking bone marrow fibroblast precursor cell migration in the heart and their differentiation towards myofibroblasts [43] (Figure 5C).

IL-13 can trigger cardiac tissue resident macrophage to display M2 anti-fibrotic phenotype [212] while IL-33, beside MCs activation via ST2, attenuates tissue-remodeling and reduces fibrosis after cardiac injury [213], by protecting cardiac fibroblasts and cardiomyocytes during inflammatory injury and hypoxia (Figure 5C). At the same time, other MCs products, such as VEGF [198], promote re-capillarization of cardiac tissue and reduce fibrosis [214] (Figure 5C).

Additionally, degranulation products have been shown to reduce fibrosis and exert cardioprotective roles [215]. MCs granules isolated from rat peritoneal fluid decreased fibrosis, enhanced survival of cardiomyocytes and increased angiogenesis after acute myocardial infarction [215]. However, few in vivo studies were able to demonstrate an anti-fibrotic role of MCs [216], while other evidence suggests irrelevant roles of MCs in cardiac fibrosis [217]. In response to increased hemodynamic load, no difference in fibrosis was observed in a MCs-deficient model mouse (C57BL/6-Kit^W-sh/W-sh^), when compared to wildtype mice [218]. Similarly, following MI, in a MC-deficient Cpa3^cre+/−^ mice, no roles for MCs in cardiac fibrosis were reported [218,219]. The contradictory conclusions point out some limitations in defining correct clinical settings and in choosing appropriate animal models [220].

Beside several pre-clinical and clinical studies placing MC as a relevant orchestrator of cardiac fibrosis, the exact mechanisms are not yet elucidated. Finally, MCs represent a valuable target for therapeutic manipulation of fibrinogenesis, thus better understanding of their involvement is urgently needed to stop and even reverse cardiac fibrosis.

## 5. Clinical Perspectives and Future Directions

Accumulating evidence indicates that myocardial fibrosis contributes to the pathogenesis of diastolic dysfunction [221,222]. This is conceivable because the structural properties of the heart are determined not only by the myocyte network, but also by interstitial connective tissue. Thus, changes in the amount and composition of the extracellular matrix should affect the diastolic properties of the LV. Ability to investigate this issue in patients has long been hampered by lack of suitable methodology, because past investigations have been restricted to evaluating cardiac fibrosis in tissue biopsies or at autopsy. The advent of cardiac magnetic resonance (CMR) has been shown to provide an accurate, non-invasive means of detecting myocardial fibrosis, due to various forms of cardiomyopathies [21,22,223,224]. Indeed, in patients with various degrees of cardiac impairment, the extent of cardiac fibrosis reliably predicts the degree of diastolic dysfunction, and of adverse outcome [225].

The obvious, important questions for future research are whether it is possible to induce regression of cardiac fibrosis, and whether this eventually translates into better prognosis for CVD patients. In spontaneously hypertensive rats (SHRs), the ACE-inhibitor lisinopril was administered for 12 weeks either as low dose that did not normalize blood pressure, or a high dose, in comparison with normotensive rats (Wistar–Kyoto (WKYs)). A regression in cardiac fibrosis was found with both doses, independent of effects of blood pressure, which was associated with normalization of LV diastolic stiffness [226]. In a following step, Brilla et al., investigated a small number of hypertensive patients (*n* = 35) with left ventricular hypertrophy and diastolic dysfunction at echocardiography. After 6 months treatment, reduction in cardiac hydroxyproline content was found, while echocardiographic parameters of diastolic dysfunction had improved [227]. These promising results need to be replicated, and expanded, in larger randomized clinical trials. However, they pave the road for future investigations in the field, the next conceptual and clinically relevant step being to be able to directly interfere with the activation of immune response.

Finally, it should also be pointed out that a low grad of fibrosis, together with the initial inflammatory events, are necessary events in reparative processes, such as the scar formation in the heart, following acute injuries. These considerations, altogether, further suggest that dramatically limiting inflammation in heart fibrosis could act as a double edge-sword.

## 6. Conclusions

Cardiac fibrosis has long been associated with adverse prognostic outcome in heart diseases. Therefore, shedding light on the pathogenesis and consequences of fibrotic lesions on cardiac disfunction are challenging aspects of the near future. Important issues regarding anti-fibrotic therapies and development of diagnostic and prognostic tools also need further investigation. In addition to the drugs currently used to treat heart failure, promising therapies targeting fibrosis are being developed, but they are still in the early beginning.

Important information to move forward on novel anti-fibrotic strategies may arrive by dissecting specific mechanisms switching human reparative responses in myocardial fibrosis and hear failure.

In this context, inflammation has a relevant role in instructing the immune environment necessary to initiate and subsequently promote cardiac fibrosis. The crosstalk between immunological and non-immunological tissue-resident cells in the injured heart still deserves elucidation on the major mechanisms involved and the reciprocal timing. In this complex scenario a better understanding of the time frame, sequences of events of the infiltration of the different immune cells and their actions in the injured inflammatory heart environment, still represent relevant clinical unmet needs, necessary to find the rational to better design novel therapeutic interventions to reduce cardiac fibrosis acting on inflammation.

## Abbreviations

α-SMA	Alfa-Smooth Muscle Actin
ADCC	Antibody-Dependent Cellular Cytotoxicity
CAD	Coronary Artery Diseases
CaMKII	Ca^2+^/calmodulin-dependent Protein Kinase II
CCL-x	Chemokine (C-C motif) ligand-x
CCR-x	C-C chemokine receptor type-x
CD49a	Alfa-1-Integrin
CD9	Tetraspanin
Cpa3	Carboxypeptidase A3
CRC	Colo-Rectal Cancer
CVB	Coxsackievirus group B
CVD	Cardiovascular diseases
CXCL-x	C-X-C Motif Chemokine Ligand
CX3CR-x	CX3C chemokine receptor-x
DAMPs	Damage-Associated Molecular Patterns
dNK	Decidual-natural Killer
EAM	Experimental model of acute myocarditis
ECM	Extra Cellular Matrix
EndoMT	Endothelial-Mesenchymal Transition
ErbB4	Receptor tyrosine-protein kinase ErbB-4
ERK	Extracellular-Signal-Regulated Kinase
ET-1	Endothelin-1
FAK/PTK2	Focal Adhesion Kinase
FcγRIIIa/CD16	Low affinity Immunoglobulin gamma Fc Region Receptor IIIa
FGF	Fibroblast Growth Factor
ICAM1/CD54	Intercellular Adhesion Molecule 1
IFN-γ	Interferon-gamma
IL	Interleukin
IL-R	Interleukin-Receptor
iNOS	Inducible Nitric Oxide Synthases
IRAK4/1	Interleukin-1 Receptor-Associated Kinase 4
M1	M1-like Macrophages
M2	M2-like Macrophages
MAPK	Mitogen-Activated Protein Kinase
MCP-1/CCL2	Monocyte chemoattractant protein-1
MertK	MER-Tyrosine-Protein Kinase
MI	Myocardial Infartion
MMPs	Matrix Metallo Peptidases
MRTF	Myocardin-related Transcription Factor
NADPH	Reduced Nicotinamide Adenine Dinucleotide Phosphate
NCAM/CD56	Neural Cell Adhesion Molecule
NET	Neutrophil-Extracellular- Traps
NFκB	Nuclear Factor kappa-light-chain-enhancer of activated B cells
NGAL	Neutrophil Gelatinase-Associated Lipocalin
NK	Natural Killer
NLRP3	NLR family Pyrin domain containing 3
NRG-1	Neureregulin-1
NSCLC	Non-Small-Cell Lung Carcinoma
OSM	Oncostatin-M
PAMPS	Pathogen-Associated Molecular Patterns
PDGF-x	Platelet-Derived Growth Factor -x
PI3K	Phosphoinositide-3 Kinase
RAGE	Receptor for Advanced Glycation End products
RANTES/CCL5	Regulated upon Activation, Normal T cell Expressed and Secreted
Reg3β	Regenerating islet-derived 3 beta
ROS	Reactive Oxygen Species
ST2	Suppression of Tumorigenicity 2
STAT3	Signal Transducer and Activator of Transcription 3
TGF-β	Transforming Growth Factor-beta
TIMPs	Tissue Inhibitor of Metalloproteinases
TLRs	Toll-Like Receptors
TNF-α	Tumor Necrosis Factor-alfa
TRAIL	Tumor Necrosis Factor (TNF)-Related Apoptosis Inducing Ligand
VEGF	Vascular Endothelial Growth Factor
